# Queen Victoria's Commemoration

**Published:** 1897-02-13

**Authors:** 


### The Institutional Workshop.

### QUEEN VICTORIA'S COMMEMORATION.

### PRINCE OF WALES'S HOSPITAL FUND FOR LONDON.

"The right cause, the right method, and the right man. For weeks and months we have all been asking each other, what shall we do to commemorate this year of years? It would be hard to say what scheme has not been mooted in one place or another; while here and there some minor proposal, usually of merely local interest, has become something more than a pious aspiration. But in the main the public has wisely held its hand, waiting for a responsible lead. That lead has now been given, and at a well-chosen moment, neither so far from the eventful day as to attentuate the collective enthusiasm of the response, nor so near to it as to prevent this from being given completely, on sound principles and without unseemly rush. . . . If this thing be taken up in the proper spirit, as we are convinced it will be, the time should not be far distant when one's hospital subscription will be given as automatically as one's contribution to the rates and with all the difference in cheerfulness in the world."—*Pall Mall Gazette.*

On the 16th ultimo we expressed confidence that the Prince of Wales's announcement of the Queen's belief, that due support will be given to works of mercy among the sick and suffering in selecting objects for the commemoration of her sixtieth anniversary as Queen of the British Empire, would put courage and heart into the supporters of the great voluntary charities of this country. We pointed out that they might certainly look forward to public opinion so shaping itself as to secure that the hospitals and medical institutions, as well as the great charities, will not be forgotten when London and other great centres of population finally determine the form of commemoration to which public support will be invited. When the article to which we refer was written, we were aware of active steps which were being taken by H.R.H. the Prince of Wales, and of his earnest desire to do something to permanently aid the hospitals of London by placing them on a sound financial basis. The original proposal to raise a great Endowment Fund was properly negatived as impracticable. The Prince's scheme to raise £150,000 in annual subscriptions from all classes of Londoners in amounts of 1s. a year upwards is a great scheme, which has already fired the public imagination, and will, we make no doubt, if properly managed, lead to a triumphant success. We hope that so far as the present year is concerned a free gift will be made to the hospitals, but that that gift will be accompanied by an intimation of the lines upon which the committee will propose to proceed in future years with reference to the distribution of the funds entrusted to them, so that this movement of His Royal Highness on behalf of the hospitals may tend to promote economy and efficiency, and to secure improved adminstration wherever necessary throughout the whole of the metropolitan hospital system. It was a happy idea to provide for the distribution of the present year's grants by seeking the co-operation of the Distribution Committee of the Hospital Sunday Fund. There is probably no body of men more trusted, and rightly so, by every shade of hospital opinion than the Distribution Committee. This wise decision on the part of the Prince of Wales must tend to secure the confidence of the public, who cannot fail to recognize in this step a guarantee that the moneys entrusted to H.R.H. will be carefully administered and properly distributed.

It would appear from some of the letters which have been published by our contemporaries in the lay press in regard to this scheme, that there are certain persons in London who still doubt the need of hospitals, and who are altogether ignorant of the work they do, either as to its quality, quantity, or necessity. We have a suggestion to make to every Londoner and especially every pressman in London, for, to ensure the success of the Prince of Wales's Fund, it is certain that we require a measure of personal service from every resident in the metropolis, and especially from every resident who has the opportunity of spreading a knowledge of the Prince of Wales's Fund, or of influencing his fellows to contribute at any rate a shilling per annum through it to the voluntary hospitals and convalescent institutions. Last week we spent four hours in the London Hospital at Whitechapel. When we went in we visited the casualty-room, and there found some thirty men, women, and children suffering from various injuries, the result of accidents, or from acute illness of various kinds. Having transacted our business, we returned to the surgery to find that not only were there still thirty cases waiting, but that those thirty had increased to upwards of one hundred in the four hours of our absence. We examined the various books in which the names and ailments of the patients were entered, and ascertained from the staff in charge of the casualty department, that on an average, for twelve hours a day at least, thirty people are attended to here on most days in each week. Further, there are often a great many more than thirty on Mondays and Tuesdays. Our suggestion is that every London man or woman, especially if journalists, who have sufficient intelligence to recognise the importance to the poor of the hospital system of London, should charge themselves with the duty of visiting the London Hospital on Monday or Tuesday in some week during the next month; that they should go to the casualty department when they first arrive, study its working, and see the class of cases treated, and attempt to realise from the books and from the patients the immense service which is there rendered to some of the poorest of the poor in this vast metropolis. Having done this they should ask to be taken to the out-patient department of the London Hospital, and they should there try to realise, as they can easily realise, the class of patients who go to that branch of the London Hospital, and see for themselves how poor the people are, how urgent are many of the cases, how impossible it is at present to deal with them adequately, and then to ask themselves what London would be without its hospitals, and especially without its casualty departments and hospital beds.

We make bold to say that were it not for the hospitals of London our great city must be in a state of revolution and anarchy, for the miseries of the poor without them would be past all bearing, when they suffer, as they must necessarily suffer in the more crowded and congested districts, from frequent accidents and other severe though often temporary ailments. Having spent an hour in the out-patient department, we would ask the visitor to return to the casualty department, and to compare the condition it is in on the second visit as to numbers with the appearance that it presented when they first arrived. The best hour to go to the out-patient department is from one o'clock to three, and to the casualty department at any time from ten o'clock in the morning till twelve o'clock at night. We are confident that any member of the Press, and indeed of the public, who has a genuine interest in the matter, and who desires honestly to ascertain the truth, will be welcome at the London Hospital, or, indeed, at any of the hospitals, if they accept the suggestion which we venture to make, and act upon it.

The Editors of the metropolitan Press have given generous support to the Prince of Wales's Hospital Fund for London, and we make no doubt that they would welcome "copy" of the character which may be readily obtained by the intelligent and thoughtful who will take the trouble to give a little of themselves in an attempt to realise the inestimable value of the work done by the hospitals of London, not only for the poor, but for every class of its inhabitants. The subject is topical, the occasion is important, and our knowledge of journalists assures us, that this invitation will not fail to induce many earnest men and women in that profession to study the hospital question on the spot, and to see whether they cannot, by the skilful use of their brains and pens, stir up and keep moving the feeling of brotherly love and gratitude and of appreciation for nurses, doctors, committeemen, and patients. Journalists should exert themselves so that the heart of London may be moved to worthily express, as the capital of the Empire and the greatest city in the world, its sentiment of love and gratitude to the Queen in the sixtieth year of her reign, and its appreciation of the good cause to which the Prince of Wales has determined to devote his best energies, and which he has declared to be very near his heart.

A Word to our Readers.

We have given a good deal of thought as to how The Hospital may best help the Prince of Wales's Fund. Of course, we shall be glad to afford our readers the fullest opportunity for subscribing to it, whether they be residents in London or elsewhere, seeing that everybody in the whole Empire is indebted to the work of the metropolitan hospitals, for through their agency medicine and surgery have developed, and from their portals have issued the majority of the members of the great medical profession, to whose devotion the English race owe so much. Of course, we shall be willing to open a list of subscribers, and to receive any subscriptions which may be forwarded to the Manager, The Scientific Press, 28 and 29, Southampton Street, Strand, London, W.C. We publish in another column a banker's form which may be used by all who have banking accounts either at a private joint-stock bank, or at the Cheque Bank, or at the Post Office Savings' Bank, or in a trustees' savings bank, or a deposit account at any of the stores. Anyone having money standing to their credit at any of these banks and stores may fill in the subscription form and send it to the Manager of the Scientific Press, so that their subscription may be acknowledged, and the order forwarded in due course to the treasurer of the Prince of Wales's Hospital Fund for London. There remains the further consideration as to whether any of our readers would like to have a small book in which they could get their friends or their patients to enter their names and addresses as annual subscribers to the Prince of Wales's Fund. If a considerable number of our readers are prepared to give personal service by undertaking the duty of collecting money in this way, especially if they be connected with some institution, or are working at their profession, we shall be prepared to print and issue subscription books in proper form to aid them in their efforts to awaken, amongst those who have not heretofore contributed regularly to hospitals, a sense of the importance of hospitals, and of appreciation of the happiness of doing what they can to aid the sick and suffering in this vast city of London.

---

The following is the letter of H.R.H. the Prince of Wales, which we have pleasure in publishing by request:—

Having ascertained from the Queen that she has no wish to express a preference for any one of the many proposals loyally suggested for commemorating, nationally or locally, the sixtieth year of her reign, I feel at liberty to bring to the notice of the inhabitants of the metropolis a project lying very near my heart, its object being to attach the sentiment of gratitude for the blessings which the country has enjoyed during the last sixty years to a scheme of permanent beneficence.

The finances of the hospitals of London have long been a source of anxiety and solicitude. An analysis furnished me of the audited statements of account for the year 1895 of 122 metropolitan hospitals and convalescent homes shows a deficiency of £70,000 in the ordinary receipts as compared with the ordinary expenditure, while, if we limit the figures to institutions which failed to meet their outgoings, the deficiency is increased to £102,500.

In considering how this may be remedied, I have been struck by the statement, the truth of which is placed beyond doubt, that the contributors to the funds of our hospitals number less than one in a hundred of the population. It appears to me that in this fact we may find at once an explanation of present indigence and the best hope of its relief. It is necessary to enlarge the area from which annual subscriptions are gathered. If we divide the population of the metropolitan district into two portions, and agree that one moiety is unable to contribute anything, there still remain three millions of persons, representing, say, 500,000 households. Of these, 450,000 households, at least so far as can be ascertained, do not contribute anything towards the support of hospitals. If we again assume that one-half are unwilling or unable to acknowledge either privilege or duty in this matter, an average annual subscription of no more than 10s. each from the remainder will suffice. The efforts of individual institutions, competing with one another, have not availed to enlist a large body of subscribers. I do not believe that this arises from any real indifference, but partly from the difficulty of choosing an object of interest among so many, partly from the lack of any definite opportunity for giving annual subscriptions to the cause as a whole, and partly from the feeling that small sums are not worth contributing. I am, however, confident that a combined appeal on behalf of the hospitals of London, setting forth their work in its magnitude and importance, will prove irresistible.

In that belief I have asked the co-operation of the representative committee, whose names are appended, and I propose with their assistance to invite subscriptions of 1s. per annum and upwards from all classes for "The Prince of Wales's Hospital Fund for London to commemorate the sixtieth anniversary of the Queen's reign." It will be noticed that the members of the committee are not identified as active managers with any particular hospital, neither shall we trench upon the ground occupied by the Hospital Sunday and Saturday Funds. Our attention will be concentrated upon an endeavour to secure from £100,000 to £150,000 in annual subscriptions from those who have not hitherto regularly contributed.

To this end we propose to approach, among others, the ground landlords, the railway and other companies, all large employers of labour, the private and joint-stock banks and companies, the various trade associations, and, above all, householders in the town and suburbs whose names are not found in the hospitals' lists.

I am glad to say that the promises of assistance received so far are most encouraging. Lord Rothschild has consented to be the treasurer of the fund, and the Governor and Directors of the Bank of England have placed office accommodation at our service both in the City and at their West-end branch. The Lord Mayor has promised his cordial support.

I am aware that the task of distributing the funds when collected will not be without its difficulties. It may be desirable in the future to seek the assistance of representatives chosen by the hospitals; but for the first year we shall rely upon the co-operation of the Distribution Committee of the Hospital Sunday Fund.

Finally, I venture to offer a word in general commendation of the scheme. Public opinion has shown itself upon more than one occasion, and I think wisely, in favour of the maintenance of the voluntary system for support of our hospitals, combined with an adequate system of representation of the body of subscribers in their control and management. It is obvious, however, that if these institutions are to be saved from State or parochial aid, their financial condition must be secured. We must recall the fact that apart from the purely philanthropic work carried on in relief of our sick poor, we look to the voluntary hospitals for the means of medical education and the advancement of medical science. Our hope is that by the aid of this Commemoration Fund we may be enabled to secure for these necessary institutions sufficient and permanent support.

Subscriptions of 1s. and upwards may be sent to the Treasurer, Lord Rothschild, New Court, E.C.; or to the Honorary Secretaries, the Prince of Wales's Hospital Fund for London, the Bank of England, London, E.C. It is hoped that arrangements may be possible in the near future by which the greatest facilities will be given to every Londoner to pay his subscription with the minimum of trouble to himself and the maximum of security for the fund.

ALBERT EDWARD, P.

Marlborough House, Pall Mall, S.W., February 5th.

THE GENERAL COUNCIL.

H.R.H. the Prince of Wales, President.
The Lord Lieutenant of Middlesex (the Earl of Strafford).
The Postmaster-General (the Duke of Norfolk).
The Bishop of London.
Cardinal Vaughan.
The Bishop of Stepney.
The Rev. J. Guinness Rogers.
The Chief Rabbi (the Rev. Dr. Adler).
Lord Rothschild.
Lord Rowton.
Lord Iveagh.
The President of the Hospital Sunday and Hospital Saturday Funds (the Lord Mayor).
The Chairman of the County Council (Sir Arthur Arnold).
The Chairman of the School Board for London (the Marquis of Londonderry).
The Governor of the Bank of England (Mr. Albert G. Sandemann).
The President of the Royal Society (Sir Joseph Lister, Bart.)
The President of the Royal College of Physicians (Dr. Wilks).
The President of the Royal College of Surgeons (Sir William MacCormac).
Sir Horace Farquhar, Bart., M.P. for West Marylebone.
Mr. John Aird, M.P. for Paddington.
Mr. Sydney Buxton, M.P. for Poplar.
Mr. Henry C. Burdett.
Mr. E. A. Hambro.
Mr. Julius Wernher.
Treasurer—Lord Rothschild.
Honorary Secretaries—The Right Hon. Stuart Wortley, Q.C., M.P.; Sir Savile Crossley, Bart.
Honorary Assistant Secretaries—Mr. Ian Malcolm, M.P.; Mr. J. G. Craggs, F.C.A.
Offices—The Bank of England, Threadneedle Street, London, E.C.

---

"THE HOSPITAL" LIST OF ANNUAL SUBSCRIBERS.

*Banker's Order Form.*

THE PRINCE OF WALES'S HOSPITAL FUND FOR LONDON.

